# Nutrition and lifestyle in healthy aging: the telomerase challenge

**DOI:** 10.18632/aging.100886

**Published:** 2016-01-30

**Authors:** Virginia Boccardi, Giuseppe Paolisso, Patrizia Mecocci

**Affiliations:** ^1^ Institute of Gerontology and Geriatrics, Department of Medicine, University of Perugia, Perugia, Italy; ^2^ Department of Medical, Surgical, Neurological, Aging and Metabolic Sciences, Second University of Naples, Naples, Italy

**Keywords:** nutrition, elderly, telomere, telomerase, longevity

## Abstract

Nutrition and lifestyle, known to modulate aging process and age-related diseases, might also affect telomerase activity. Short and dysfunctional telomeres rather than average telomere length are associated with longevity in animal models, and their rescue by telomerase maybe sufficient to restore cell and organismal viability. Improving telomerase activation in stem cells and potentially in other cells by diet and lifestyle interventions may represent an intriguing way to promote health-span in humans.

Aging is defined as the progressive decline in physiological functions which leads to increased vulnerability to diseases and death [1]. This is a universal process underlying by many mechanisms and different pathways, whose burden rises to three different phenotypes: normal aging, accelerated aging and successful aging [2]. Despite variability among definitions, “successful aging” is as a multidimensional process encompassing major chronic diseases, major impairments in cognitive, in physical function and sustained engagement in social and productive activities [2,3]. However, reaching old age in good health is not just a “fate effect” but the result of a complex interweaving between environmental and genetic factors [4]. Studies conducted in twins have estimated that approximately 20-30% of an individual's lifespan is related to genetics, while the rest is due to individual behaviors and environmental factors [5,6]. In this contest, nutrition and lifestyle are the most important contributors to longevity and healthy aging [7-11]. Follow a diet rich in vegetables, fruits, nuts, olive oil, fish, a small amount of red wine and exercise at least 20 minutes a day three times a week, avoiding obesity, smoke and alcohol, represents the working recipe for long and healthy life. Many mechanisms and pathways underlie nutrition, lifestyle and longevity including telomere length modulation [12-15].

Telomeres are long sequences of nucleotides at the end of our chromosomes, forming with specific proteins complex, an “end caps” which preserve genome stability and lead a cell to correctly divide [16-18]. Telomeres have been compared with the plastic tips on shoelaces, since they are able to keep chromosome ends from fraying and fusion to each other, which would destroy or interfere genetic information. At each cell division or replication event, telomeres lose some of their length and when they get too short, the cell is no longer able to divide becoming "senescent" [19]. This shortening process triggers a sustaining damage response scrambling with cell health leading to disease risk and cell death [20]. In 1962, Leonard Hayflick revolutionized cell biology when he developed a telomere theory known as the “Hayflick limit”, which places the maximum potential lifespan of humans at 120 years, the time at which too many cells with extremely short telomeres can no longer replicate and divide [21,22]. Fifty years later, new science came out opening the door to maximizing our genetic potential. In fact, published data suggested that extremely short or dysfunctional telomeres can be repaired by the enzyme “*telomerase”*, which working as a reverse transcriptase, adds nucleotides at the end of each chromosome promoting its stability [22,23]. In 2009, Blackburn, Greider and Szostak received the Nobel Prize for the discovery of “how chromosomes are protected by telomeres and the enzyme telomerase”. These discoveries had a great impact within the scientific community, supporting that aging can be potentially delayed by telomerase activation and telomere erosion rate reduction.

In contrast to stem cells which constitutively express low levels of telomerase, normal somatic human cells repress its expression immediately after birth [24-27]. Thus, for a long time, telomere length has been considered as an indicator of cellular senescence, and a potential biomarker of human aging, but studies supporting this role are still contradictory and inconclusive [22,28,29]. More recent genetic studies in animal models have demonstrated that short telomeres rather than average telomere length are associated with age-related diseases and, their rescue by telomerase is sufficient to restore cell and organismal viability [30,31]. In humans, circulating telomerase activity rather than telomeres length is inversely associated with the major cardiovascular disease risk factors [32]. Thus, another concept is coming up, the “telomere stability”, a quite different concept from telomere length. For example, patients with Alzheimer's disease do not invariably have shorter telomeres, but their telomeres have significant signs of dysfunction [33-38]. Improving the activity of telomerase enzyme -that can add length back to shorter telomeres, and, in the meantime, protect longer telomeres to ensure stability- seems a way to actually turn back the biological clock. Telomerase has also extra-telomeric functions influencing various essential cellular processes, such as gene expression, signaling pathways, mitochondrial function as well as cell survival and stress resistance [40,41]. Therefore, the presence of active telomerase in stem cells, and potentially in all cells, may be helpful for longevity and good health.

Lifestyle factors known to modulate aging and age-related diseases might also affect telomerase activity. Obesity [42], insulin resistance [43,44], and cardio-vascular disease processes [45,46], which are related to oxidative stress and inflammation, have all been linked to shorter telomeres. Smoking, exposure to pollution, lower physical activity, psychological stress, and unhealthy diet significantly increase the oxidative burden and the rate of telomere shortening [47-53]. So, what a better way to counteract the “biological clock” by reactivating telomerase trough diet and lifestyle interventions? There is a recent paper showing that with intensive lifestyle modification, with a low fat diet, regular physical activity, and mental stress reduction (by yoga and meditation), telomerase activity increases significantly in peripheral blood mononuclear cell (PBMC) [54]. Again, people living in the Mediterranean countries have longer and healthier life as compared with people living in other industrialized countries, and we previously demonstrated that they have also claim longer telomeres and higher telomerase activity in PBMC [55]. It is still unclear if there is a single nutrient or a factor responsible of Mediterranean diet anti-aging properties or the whole, single ingredient foods and lifestyle are the key to “healthspan”.

Today, researchers are struggling to find a compound or an “elixir” for long life, while common people are taking dietary supplements with the intent to preserve mental, physical, and emotional health into old age. Most dietary supplement programs include combinations of vitamins, antioxidants, and other constituents, some of which have been shown to have significant health benefits in controlled clinical studies. Specific nutrients provide all the necessary building blocks to support telomere health and extend lifespan. This is the case of folate [56,57], vitamins (B, D, E, C) [58] zinc [59] and polyphenol compounds such as resveratrol [60], grape seed extract and curcumin [61]. Several foods -such as tuna, salmon, herring, mackerel, halibut, anchovies, cat-fish, grouper, flounder, flax seeds, sesame seeds, kiwi, black raspberries, green tea, broccoli, sprouts, red grapes, tomatoes, olive fruit- are a good source of antioxidants. These, combined with a Mediterranean type of diet containing fruits, vegetables and whole grains would help protect our chromosome ends [62-70].

In conclusion, what we eat, how we eat and how much we eat, together with lifestyle significantly, can affect our telomerase/telomere system with a great impact on healthspan. “*Similes cum similibus curantur*” and in nature is still hidden the secret of healthy and long life whereas telomerase could represent the distinctive target.

## References

[1] López-Otín C, Blasco MA, Partridge L, Serrano M, Kroemer G (2013). The hallmarks of aging. Cell.

[2] Bowling A, Dieppe P (2005). What is successful ageing and who should define it?. BMJ.

[3] Martin P, Kelly N, Kahana B, Kahana E, Willcox BJ, Willcox DC, Poon LW7 (2015). Defining successful aging: a tangible or elusive concept?. Gerontologist.

[4] Sharpless NE, DePinho RA (2007). How stem cells age and why this makes us grow old. Nat Rev Mol Cell Biol.

[5] Sebastiani P, Perls TT (2012). The genetics of extreme longevity: lessons from the new England centenarian study. Front Genet.

[6] Herskind AM, Mcgue M, Holm NV, Sorensen TI, Harvald B, Vaupel JW (1996). The heritability of human longevity: a population-based study of 2872 Danish twin pairs born 1870-1900. Hum. Genet..

[7] Kiefte-de Jong JC, Mathers JC, Franco OH (2014). Nutrition and healthy ageing: the key ingredients. Proc Nutr Soc.

[8] Vasto S, Barera A, Rizzo C, Di Carlo M, Caruso C, Panotopoulos G (2014). Mediterranean diet and longevity: an example of nutraceuticals?. Curr Vasc Pharmacol.

[9] Hausman DB, Fischer JG, Johnson MA (2011). Nutrition in centenarians. Maturitas.

[10] Cannella C, Savina C, Donini LM (2009). Nutrition, longevity and behavior. Arch Gerontol Geriatr.

[11] Bengmark S (2006). Impact of nutrition on ageing and disease. Curr Opin Clin Nutr Metab Care.

[12] Paul L (2011). Diet, nutrition and telomere length. J Nutr Biochem.

[13] Lee JY, Jun NR, Yoon D, Shin C, Baik I (2015). Association between dietary patterns in the remote past and telomere length. Eur J Clin Nutr.

[14] Sun Q, Shi L, Prescott J, Chiuve SE, Hu FB, De Vivo I, Stampfer MJ, Franks PW, Manson JE, Rexrode KM (2012). Healthy lifestyle and leukocyte telomere length in U.S. women. PLoS One.

[15] Cassidy A, De Vivo I, Liu Y, Han J, Prescott J, Hunter DJ, Rimm EB (2010). Associations between diet, lifestyle factors, and telomere length in women. Am J Clin Nutr.

[16] Sfeir AJ, Chai W, Shay JW, Wright WE (2005). Telomere end processing: the terminal nucleotides of human chromosomes. Mol. Cell.

[17] de Lange T (2002). Protection of mammalian telomeres. Oncogene.

[18] Baird DM, Kipling D (2004). The extent and significance of telomere loss with age. Ann NY Acad Sci.

[19] Campisi J, d'Adda di Fagagna F (2007). Cellular senescence: when bad things happen to good cells. Nat. Rev. Mol. Cell Biol..

[20] Tacutu R, Budovsky A, Yanai H, Fraifeld VE (2011). Molecular links between cellular senescence, longevity and age related diseases - a systems biology perspective. Aging (Albany NY).

[21] Hayflick L (1976). The cell biology of human aging. The New England Journal of Medicine.

[22] Blackburn EH (2000). Telomere states and cell fates. Nature.

[23] Blackburn EH (2001). Switching and signaling at the telomere. Cell.

[24] Feng J, Funk WD, Wang SS, Weinrich SL, Avilion AA, Chiu CP, Adams RR, Chang E, Allsopp RC, Yu J, Le S, West MD, Harley CB, Andrews WH, Greider CW, Villeponteau B (1995). The RNA component of human telomerase. Science.

[25] Wright WE, Piatyszek MA, Rainey WE, Byrd W, Shay JW (1996). Telomerase activity in human germline and embryonic tissues and cells. Dev. Genet..

[26] Shay JW, Bacchetti S (1997). A survey of telomerase activity in human cancer. Eur. J. Cancer.

[27] Masutomi K, Yu EY, Khurts S, Ben-Porath I, Currier JL, Metz GB, Brooks MW, Kaneko S, Murakami S, DeCaprio JA, Weinberg RA, Stewart SA, Hahn WC (2003). Telomerase maintains telomere structure in normal human cells. Cell.

[28] Sanders JL, Newman AB (2013). Telomere Length in Epidemiology: A Biomarker of Aging, Age-Related Disease, Both, or Neither?. Epidemiol Rev.

[29] Simons MJ (2015). Questioning causal involvement of telomeres in aging. Ageing Res Rev.

[30] Vera E, Bernardes de Jesus B, Foronda M, Flores JM, Blasco MA (2012). The rate of increase of short telomeres predicts longevity in mammals. Cell Rep.

[31] Calado RT, Dumitriu B (2013). Telomere dynamics in mice and humans. Semin Hematol.

[32] Epel ES, Lin J, Wilhelm FH, Wolkowitz OM, Cawthon R, Adler NE, Dolbier C, Mendes WB, Blackburn EH (2006). Cell aging in relation to stress arousal and cardiovascular disease risk factors. Psychoneuroendocrinology.

[33] Panossian LA, Porter VR, Valenzuela HF, Zhu X, Reback E, Masterman D, Cummings JL, Effros RB (2003). Telomere shortening in T cells correlates with Alzheimer's disease status. Neurobiol Aging.

[34] Honig LS, Schupf N, Lee JH, Tang MX, Mayeux R (2006). Shorter telomeres are associated with mortality in those with APOE epsilon4 and dementia. Ann Neurol.

[35] Thomas P, O'Callaghan NJ, Fenech M (2008). Telomere length in white blood cells, buccal cells and brain tissue and its variation with ageing and Alzheimer's disease. Mech Ageing Dev.

[36] Hochstrasser T, Marksteiner J, Humpel C (2012). Telomere length is age-dependent and reduced in monocytes of Alzheimer patients. Exp. Gerontol..

[37] Cai Z, Yan LJ, Ratka A (2013). Telomere shortening and Alzheimer's disease. Neuromolecular Med.

[38] Boccardi V, Pelini L, Ercolani S, Ruggiero C, Mecocci P (2015). From cellular senescence to Alzheimer's disease: The role of telomere shortening. Ageing Res Rev.

[39] Saretzki G (2014). Extra-telomeric functions of human telomerase: cancer, mitochondria and oxidative stress. Curr Pharm Des.

[40] Martínez P, Blasco MA (2011). Telomeric and extra-telomeric roles for telomerase and the telomere-binding proteins. Nat Rev Cancer.

[41] Saretzki G (2009). Telomerase, mitochondria and oxidative stress. Exp Gerontol.

[42] Mundstock E, Sarria EE, Zatti H, Mattos Louzada F, Kich Grun L, Herbert Jones M, Guma FT, Mazzola In Memoriam J, Epifanio M, Stein RT, Barbé-Tuana FM, Mattiello R (2015). Effect of obesity on telomere length: Systematic review and meta-analysis. Obesity (Silver Spring).

[43] Strazhesko I, Tkacheva O, Boytsov S, Akasheva D, Dudinskaya E, Vygodin V, Skvortsov D, Nilsson P (2015). Association of Insulin Resistance, Arterial Stiffness and Telomere Length in Adults Free of Cardiovascular Diseases. PLoS One.

[44] Demissie S, Levy D, Benjamin EJ, Cupples LA, Gardner JP, Herbert A (2006). Insulin resistance, oxidative stress, hypertension, and leukocyte telomere length in men from the Framingham Heart Study. Aging Cell.

[45] Haycock PC, Heydon EE, Kaptoge S, Butterworth AS, Thompson A, Willeit P (2014). Leucocyte telomere length and risk of cardiovascular disease: systematic review and meta-analysis. BMJ.

[46] Zietzer A, Hillmeister P (2014). Leucocyte telomere length as marker for cardiovascular ageing. Acta Physiol (Oxf).

[47] Barrett JH, Iles MM, Dunning AM, Pooley KA (2015). Telomere length and common disease: study design and analytical challenges. Hum Genet.

[48] Cherkas LF, Hunkin JL, Kato BS, Richards JB, Gardner JP, Surdulescu GL, Kimura M, Lu X, Spector TD, Aviv A (2008). The association between physical activity in leisure time and leukocyte telomere length. Arch Intern Med.

[49] Cassidy A, De Vivo I, Liu Y, Han J, Prescott J, Hunter DJ, Rimm EB (2010). Associations between diet, lifestyle factors, and telomere length in women. Am J Clin Nutr.

[50] Mirabello L, Huang WY, Wong JY, Chatterjee N, Reding D, Crawford ED, De Vivo I, Hayes RB, Savage SA (2009). The association between leukocyte telomere length and cigarette smoking, dietary and physical variables, and risk of prostate cancer. Aging Cell.

[51] Strandberg TE, Saijonmaa O, Tilvis RS, Pitkälä KH, Strandberg AY, Miettinen TA, Fyhrquist F (2011). Association of telomere length in older men with mortality and midlife body mass index and smoking. J Gerontol A Biol Sci Med Sci.

[52] LaRocca TJ, Seals DR, Pierce GL (2010). Leukocyte telomere length is preserved with aging in endurance exercise-trained adults and related to maximal aerobic capacity. Mech Ageing Dev.

[53] Chen W, Kimura M, Kim S, Cao X, Srinivasan SR, Berenson GS, Kark JD, Aviv A (2011). Longitudinal versus cross-sectional evaluations of leukocyte telomere length dynamics: age-dependent telomere shortening is the rule. J Gerontol A Biol Sci Med Sci.

[54] Ornish D, Lin J, Chan JM, Epel E, Kemp C, Weidner G, Marlin R, Frenda SJ, Magbanua MJ, Daubenmier J, Estay I, Hills NK, Chainani-Wu N, Carroll PR, Blackburn EH (2013). Effect of comprehensive lifestyle changes on telomerase activity and telomere length in men with biopsy-proven low-risk prostate cancer: 5-year follow-up of a descriptive pilot study. Lancet Oncol.

[55] Boccardi V, Esposito A, Rizzo MR, Marfella R, Barbieri M, Paolisso G (2013). Mediterranean diet, telomere maintenance and health status among elderly. PLoS One.

[56] Fenech M (2012). Folate (vitamin B9) and vitamin B12 and their function in the maintenance of nuclear and mitochondrial genome integrity. Mutat Res.

[57] Moores CJ, Fenech M, O'Callaghan NJ (2011). Telomere dynamics: the influence of folate and DNA methylation. Ann N Y Acad Sci.

[58] Sen A, Marsche G, Freudenberger P, Schallert M, Toeglhofer AM, Nagl C, Schmidt R, Launer LJ, Schmidt H (2014). Association between higher plasma lutein, zeaxanthin, and vitamin C concentrations and longer telomere length: results of the Austrian Stroke Prevention Study. J Am Geriatr Soc.

[59] Sharif R, Thomas P, Zalewski P, Fenech M (2012). The role of zinc in genomic stability. Mutat Res.

[60] Jayasena T, Poljak A, Smythe G, Braidy N, Münch G, Sachdev P (2013). The role of polyphenols in the modulation of sirtuins and other pathways involved in Alzheimer's disease. Ageing Res Rev.

[61] Thomas P, Wang YJ, Zhong JH, Kosaraju S, O'Callaghan NJ, Zhou XF, Fenech M (2009). Grape seed polyphenols and curcumin reduce genomic instability events in a transgenic mouse model for Alzheimer's disease. Mutat Res.

[62] Sohal RS, Weindruch R (1996). Oxidative stress, caloric restriction, and aging. Science.

[63] Assmann KE, Lassale C, Andreeva VA, Jeandel C, Hercberg S, Galan P, Kesse-Guyot E (2015). A Healthy Dietary Pattern at Midlife, Combined with a Regulated Energy Intake, Is Related to Increased Odds for Healthy Aging. J Nutr.

[64] de Cabo R, Carmona-Gutierrez D, Bernier M, Hall MN, Madeo F (2014). The search for antiaging interventions: from elixirs to fasting regimens. Cell.

[65] Dernini S, Berry EM (2015). Mediterranean Diet: From a Healthy Diet to a Sustainable Dietary Pattern. Front Nutr.

[66] Pérez-López FR, Chedraui P, Haya J, Cuadros JL (2009). Effects of the Mediterranean diet on longevity and age-related morbid conditions. Maturitas.

[67] Roman B, Carta L, Martínez-González MA, Serra-Majem L (2008). Effectiveness of the Mediterranean diet in the elderly. Clin Interv Aging.

[68] Ross SM (2015). The traditional Mediterranean diet: an ancient prescription for health and longevity. Holist Nurs Pract.

[69] Mathers JC (2015). Impact of nutrition on the ageing process. Br J Nutr.

[70] Gupta C, Prakash D (2014). Nutraceuticals for geriatrics. J Tradit Complement Med.

